# Cartilage hair hypoplasia: characteristics and orthopaedic manifestations

**DOI:** 10.1007/s11832-015-0646-z

**Published:** 2015-03-13

**Authors:** Patrick Riley, Dennis S. Weiner, Bonnie Leighley, David Jonah, D. Holmes Morton, Kevin A. Strauss, Michael B. Bober, Martin S. Dicintio

**Affiliations:** 1 Department of Pediatric Orthopaedic Surgery, Akron Children’s Hospital, Akron, OH 44308 USA; 2 Akron Children’s Hospital, Northeast Ohio Medical University, Akron, OH 44308 USA; 3 Regional Skeletal Dysplasia Clinic, Akron Children’s Hospital, Akron, OH 44308 USA; 4 Little People’s Research Fund, Baltimore, MD 21228 USA; 5 Clinic for Special Children, Strasburg, PA 17579 USA; 6 Skeletal Dysplasia Program, Alfred I. duPont Hospital for Children, Wilmington, DE 19803 USA; 7 300 Locust Street, Ste. 250, Akron, OH 44302-1821 USA

**Keywords:** Pathoanatomy, McKusick-type metaphyseal chondrodysplasia, Cartilage hair hypoplasia

## Abstract

**Purpose:**

Cartilage hair hypoplasia (CHH) is a rare metaphyseal chondrodysplasia characterized by short stature and short limbs, found primarily in Amish and Finnish populations. Cartilage hair hypoplasia is caused by mutations in the RMRP gene located on chromosome 9p13.3. The disorder has several characteristic orthopaedic manifestations, including joint laxity, limited elbow extension, ankle varus, and genu varum. Immunodeficiency is of concern in most cases. Although patients exhibit orthopaedic problems, the orthopaedic literature on CHH patients is scant at best. The objective of this study was to characterize the orthopaedic manifestations of CHH based on the authors’ unique access to the largest collection of CHH patients ever reported.

**Methods:**

The authors examined charts and/or radiographs in 135 cases of CHH. We analyzed the orthopaedic manifestations to better characterize and further understand the orthopaedic surgeon’s role in this disorder. In addition to describing the clinical characteristics, we report on our surgical experience in caring for CHH patients.

**Results:**

Genu varum, with or without knee pain, is the most common reason a patient with CHH will seek orthopaedic consultation. Of the cases reviewed, 32 patients had undergone surgery, most commonly to correct genu varum.

**Conclusion:**

This paper characterizes the orthopaedic manifestations of CHH. Characterizing this condition in the orthopaedic literature will likely assist orthopaedic surgeons in establishing a correct diagnosis and appreciating the orthopaedic manifestations. It is important that the accompanying medical conditions are appreciated and evaluated.

**Electronic supplementary material:**

The online version of this article (doi:10.1007/s11832-015-0646-z) contains supplementary material, which is available to authorized users.

## Introduction

McKusick et al. [1], in an extensive study of the Old Order Amish community in eastern Pennsylvania, clearly elucidated in 77 cases a distinct type of short-limb dwarfism termed cartilage hair hypoplasia, also known as McKusick metaphyseal chondrodysplasia. McKusick presented evidence that this disorder was inherited as an autosomal recessive with a frequency of 1–2 per thousand live births in the Old Order Amish [2].

Cartilage hair hypoplasia (CHH) has been reported in many countries and many peoples outside the Amish community [3–14]. The largest population of cases outside the Amish community is found in Finland, where Mäkitie in 1992 described the incidence as roughly 1:23,000 live births [15]. Mäkitie and colleagues, in several excellent publications, have described several associations of CHH beyond the presence of chondrodysplasia [5, 16–24]. CHH is associated with a broad spectrum of mild-to-moderate, cell-mediated immunodysfunction, including occasionally severe combined immune deficiency, neutropenia, lymphopenia, disordered erythrogenesis, and a predisposition to lymphoma [19–22, 25–37]. Recurring infections are commonly seen in 60 % of all patients, likely linked to the immunodeficiency. The severity is reflected in a report by Rider et al. [38] in which 32 % of their patients (25) had recurrent infections, with two requiring bone marrow transplantation [39–41]. Individuals affected with CHH can have marked impairment of T-lymphocyte function due to an intrinsic defect in cell proliferation. Beta cells and fibroblasts also show defective proliferation. Morbidity and mortality to varicella is clearly increased in CHH, but there is no apparent increase in susceptibility to severe or fatal infections with other viruses. Morbidity and mortality are directly related to immune dysfunction. The presence of infections, particularly in the first 2 years of life in children, is increased in frequency and hematologic malignancy is increased in adults. Mäkitie et al. [23] in 1999 reported an increased incidence of non-Hodgkin lymphoma and an excess risk of basal cell carcinoma in CHH. Others have reported similar findings, most commonly non-Hodgkin lymphoma [22, 23, 35, 36, 42–44]. Anemia is also commonly encountered and significantly correlated with the severity of immunodeficiency and growth failure.

It has been clearly documented that CHH is caused by mutations in the RMRP (RNA component of mitochondrial RNA processing endoribonuclease) gene located on chromosome 9p13–p12 and more recently narrowed to chromosome 9p13.3 [15, 16, 38, 45–60]. The RMRP 70A→G mutation is the most frequent ancestral mutation seen in Finnish and Old Order Amish people.

Patients with CHH are classically short at birth and the shortening may increase in the first 2 years of life. A very weak or absent pubertal growth spurt has been reported by Mäkitie.

**Fig. 1:**
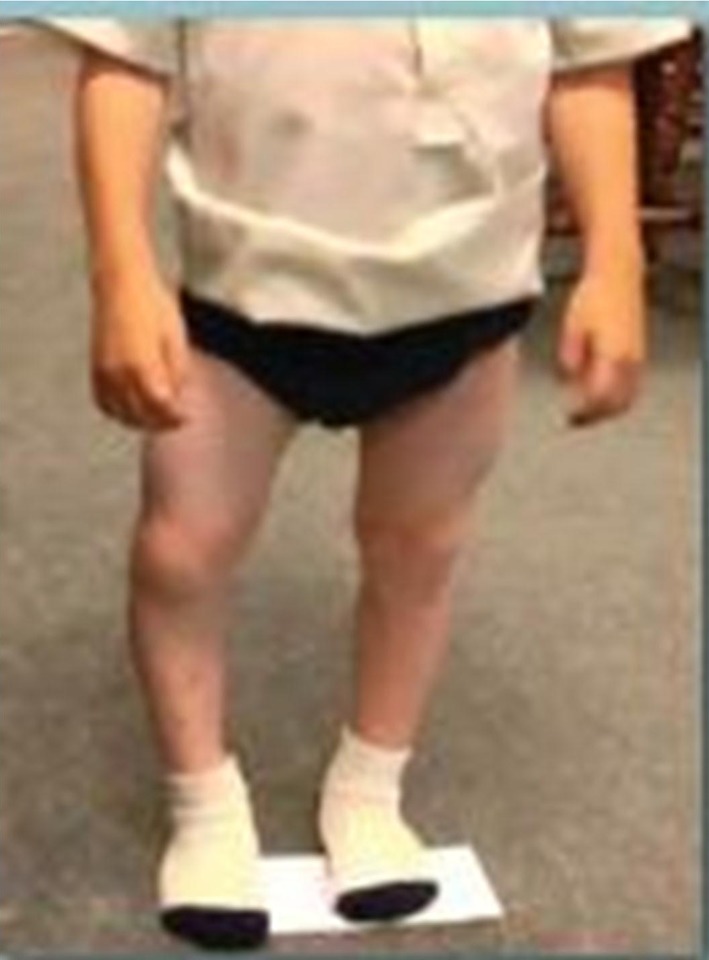
Frontal view demonstrating bowing of right femur and tibia in patient with cartilage hair hypoplasia

Typically, the patients present with fine, sparse, light-colored, “silky” short hair that breaks easily. McKusick et al. [1, 2] and Coupe and Lowry [61] examined the hair grossly, microscopically, and in diameter. The hair has been demonstrated to be diminished in caliber and lacks a pigmented core, a finding also observed by van der Burgt et al. [3]. The hands are short and “pudgy” with marked excessive joint laxity, particularly of the metacarpal, phalangeal, and interphalangeal joints [1, 62]. The elbows typically lack full extension. Bowing of the lower extremities and disproportionate length of the fibula compared to the shortened tibia is consistent, although unproven as the cause of the ankle varus, resulting in ankle varus from relative fibular overgrowth (Fig. 1).

Mäkitie et al. reported that the median adult height was 131.1 cm (range 110.7–149.0 cm) for males and 122.5 cm (range 103.7–137.4 cm) for females [18]. The radius and ulna are commonly shorter than the humerus and the femur shorter than the tibia, with the tibia being more severely involved than the fibula.

Several nonskeletal issues are of considerable importance in association with CHH. In the McKusick series, a few patients had evidence of aganglionic colon, two patients died of varicella, and three others had had virulent infections with varicella. Intestinal malabsorption and Hirschsprung disease has been reported in association with CHH [1, 63]. Mäkitie et al. [24] estimated the incidence of these two disorders as occurring in 9 % of the patients. Prominence of the upper sternal region has been mentioned in the literature but is of no functional significance, but can be useful in diagnosis.

**Fig. 2:**
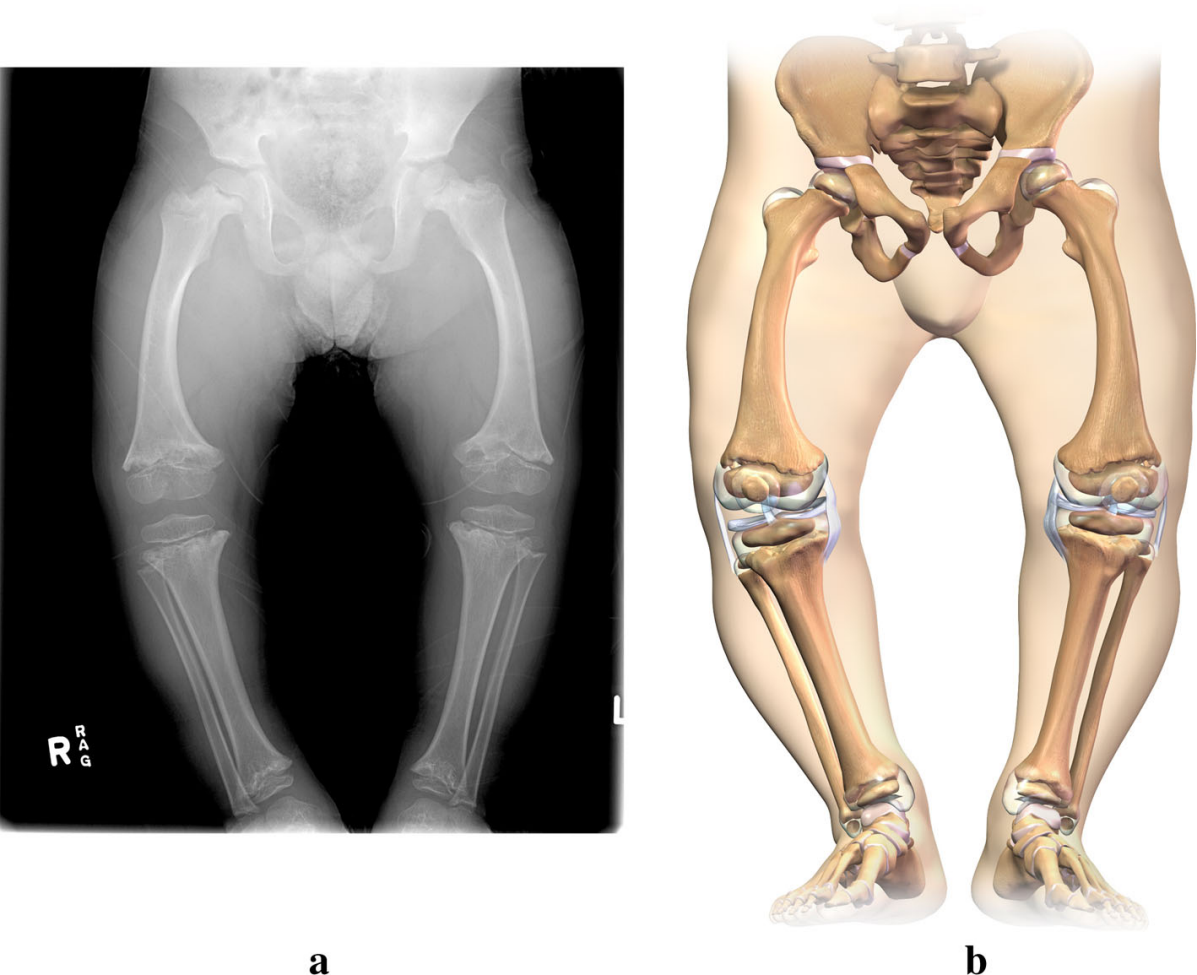
**a** Anteroposterior radiograph of the lower extremity in cartilage hair hypoplasia demonstrating bowing. **b** Illustration depicting the osseous and cartilaginous deformities of lower extremities in cartilage hair hypoplasia

Typically, the ossification centers of the epiphyses are radiographically normal. Metaphyseal abnormalities reflecting the chondrodysplasia are manifested by flaring, cupping, marginal serration, fragmentation, and scalloping of the metaphyses of the tubular bones, most particularly seen at the knee [1, 3, 17, 64–66] (Fig. 2a, b).

The hips are typically spared these findings. There are irregular cystic radiolucencies in the metaphyses with extension into the diaphyses. Abnormal epiphyseal shape occurs, seemingly as a consequence of the deformed metaphyseal region. There is marked shortening of the metacarpals, metatarsals, and phalanges, with metaphyseal cupping and “cone-shaped” epiphyses. Costochondral junctions have reflected mild flaring of the lower ribcage, slightly anterior angulated sternum, and lumbar lordosis. There is commonly delay in appearance of the proximal femoral epiphyses but absence of the proximal femoral metaphyseal changes that are typical at the knee. The entire metaphyseal region is widened and flared at the knee and ankle. The skull and spine are normal. Ray and Dorst [66] also reported mild-to-moderate C1 and C2 subluxation. Mäkitie et al. [17], however, reported in 1992 that none of 26 patients had cervical subluxation. Lachman [67] mentioned that several patients showed forward subluxation of C1 and C2 in flexion (lateral C-spine X-rays taken in neutral flexion and extension).

Mäkitie et al. demonstrated that the growth retardation in CHH correlates well with the severity of the metaphyseal changes [18, 68]. Ray and Dorst [66] reported that one-third of elbows showed lateral subluxation or dislocation of the radial head. Histologic examination was performed in one of McKusick’s cases and demonstrated paucity of cartilage cells and deficiency in columnar organization that was interpreted as cartilage hypoplasia. We could find no infrastructural histopathologic information in the literature to date. Prenatal diagnosis has been reported [69, 70].

A summary of the orthopaedic abnormalities that are typical in this disorder comprises significant joint laxity, particularly of the hands and feet, limited elbow extension, bowing of the lower leg and thigh, increased lumbar lordosis, varus deformity of the ankle due to fibular overgrowth, occasional atlantoaxial radiographic hypermobility, and scoliosis that rarely requires orthopaedic treatment.

### Material: data

A unique opportunity was afforded the authors to access data on a large number of patients through clinical examinations, records, and radiographs of the largest accumulation of CHH cases so far encountered in North America. Approval from our institutional review board was obtained. Information was obtained from the files of the senior author (D.S.W.) on 135 patients with CHH comprising 63 males and 72 females: 65 Old Order Amish and 70 non-Amish patients. Documentation of diagnosis was obtained by clinical examination (26 patients), radiographic examination (82 patients), or genetic diagnosis (27 patients). Complete physical examination was available on 75 patients and the remainder was included based on radiographic assessment and/or genetic documentation.

For the purpose of this paper, we have arbitrarily divided the orthopaedic characteristics of CHH into typical (found in over 90 % of cases), frequent (50 % or more), occasional (<50 %), and rare. Radiographic abnormalities associated with CHH were similarly separated by frequency.

## Results

**Fig. 3:**
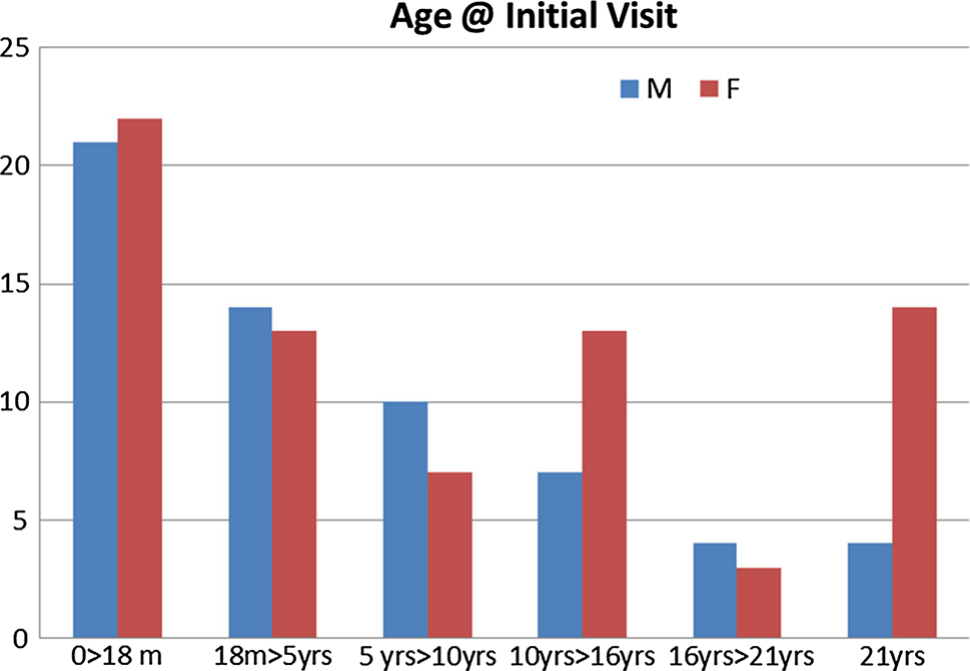
Patient age distribution at initial visit (*m* months, *yrs* years, *M* male, *F* female)

The age distribution of the patients at initial visit is shown in Fig. 3. The clinical findings seen in our population of 135 patients are recorded in Table 1 in order of their frequency. Likewise, in Table 2, radiographic features encountered are presented in accordance with their relative frequency. In our series, 27 % of the cases where radiographs were reviewed (19/71) showed coxa vara radiographically.

**Table 1:** Frequency of clinical findings in CHH

Clinical manifestation	Cases/patients reviewed	% Occurrence	Frequency
Short-limbed disproportionate dwarfism	75/75	100	Typical
Varus deformity of femur	77/83	93	Typical
Short pudgy hands with marked joint laxity	75/75	100	Typical
Varus deformity of tibia	84/96	88	Frequent
Diminished elbow extension	56/69	81	Frequent
Ankle varus deformity	85/96	87	Frequent

**Table 2:** Frequency of radiographic findings in CHH

Radiographic manifestation	Cases/patients reviewed	% Occurrence	Frequency
Disproportionate short-limb dwarfism	75/75	100	Typical
Metaphyseal abnormalities of femur and tibia	75/75	100	Typical
Shortening of the metacarpals and phalanges	75/75	100	Typical
Genu varum	85/96	87	Frequent
Coxa vara	19/71	27	Occasional

Several surgical procedures were performed on patients with angular and/or rotational deformity. In this series of patients, roughly 43 % had undergone surgical realignment of the lower extremities. The ages at surgery ranged from 2 to 27 years, with an average of 11.7 years and mean of 14.5 years. We did not encounter in our patients any cases that required cervical surgical stabilization (12 cases with lateral view of C-spine in neutral flexion and extension).

## Discussion

It is interesting that over two-thirds of the patients in McKusick’s study derive ancestry from one John Miller who was married to Catherine Hochstetler. Over 70 % of the patients trace their ancestry to Jacob Hochstetler, a relative of Catherine. Eighty percent of parents of the 50 affected patients studied by McKusick were descended from either Jacob or Catherine Hochstetler, who immigrated to the United States in the mid-1700s. Amish migration has resulted in many cases seen in other states, particularly in Ohio at present [1, 2].

The orthopaedic manifestations that have clinical significance are related to the metaphyseal chondrodysplasia resulting in bowing, angulation, and rotational deformities. Femoral bowing and coxa vara are occasionally severe enough to warrant surgical intervention by osteotomy and realignment. In the lower leg, the shortening and bowing of the tibia and the ankle varus appears directly related to the relative overgrowth of the fibula.

It would appear that the loss of elbow extension, although typically seen, is most likely related to radial head subluxation or dislocation [27]. This is probably the best explanation, inasmuch as the other joints generally demonstrate increased laxity. Although typically there is lack of elbow extension, we have not encountered a single patient who had any issues of consequence with that loss of motion.

The most common mutation in the RMRP gene located on chromosome 9p13.3 is identical in the Old Order Amish population and the Finnish population. Although C1-2 subluxation has been described, we have not, in our series, encountered any cases that have required stabilization nor have we seen any cases of scoliosis that required orthopaedic management.

**Fig. 4:**
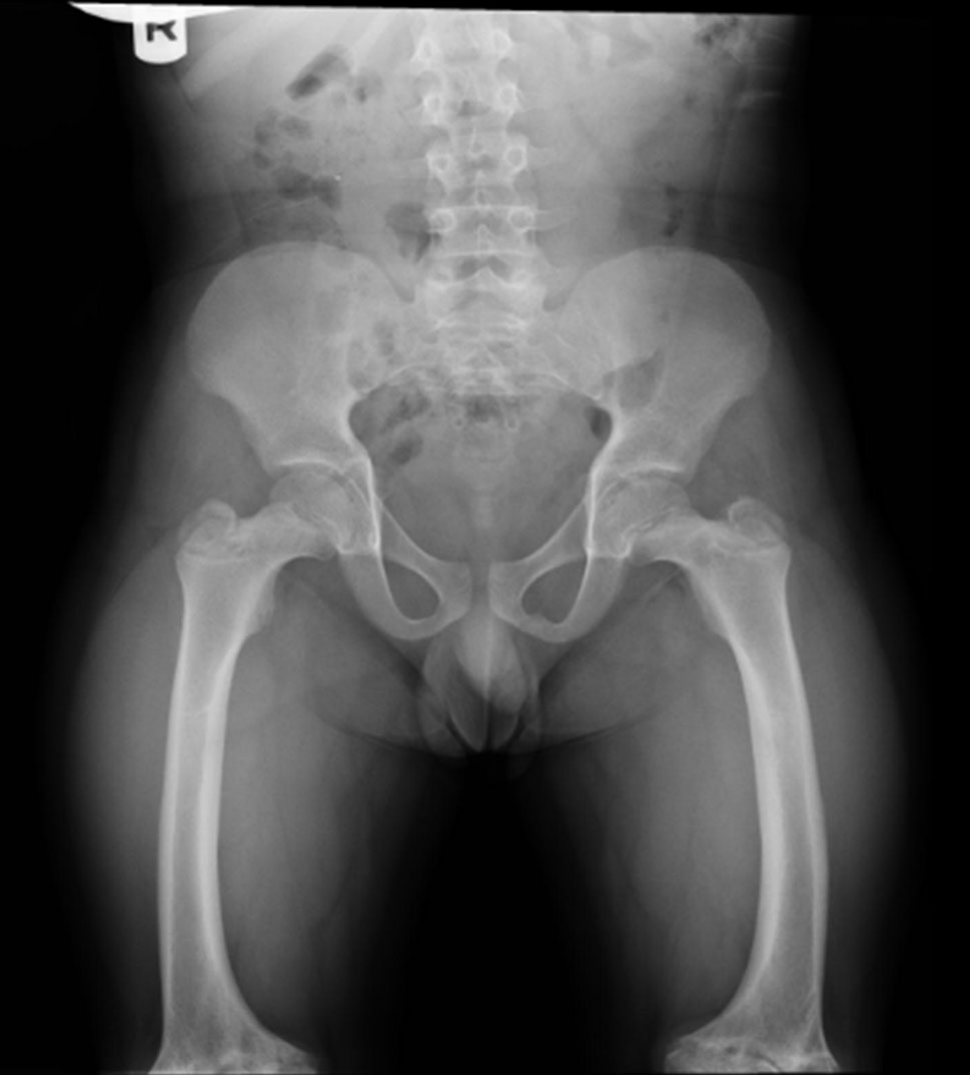
Anteroposterior radiograph demonstrating coxa vara and femoral bowing

Coxa vara is likely due to a combination of relative discrepancy in growth of the upper femoral physis to trochanteric growth (slower proximal femoral growth plate in relation to trochanter and neck) combined with bowing and shortening of the femur, although this is admittedly speculative (Fig. 4). The severity of the bowing of the femur and tibia appears to correlate with the extent of metaphyseal radiographic changes.

**Fig. 5:**
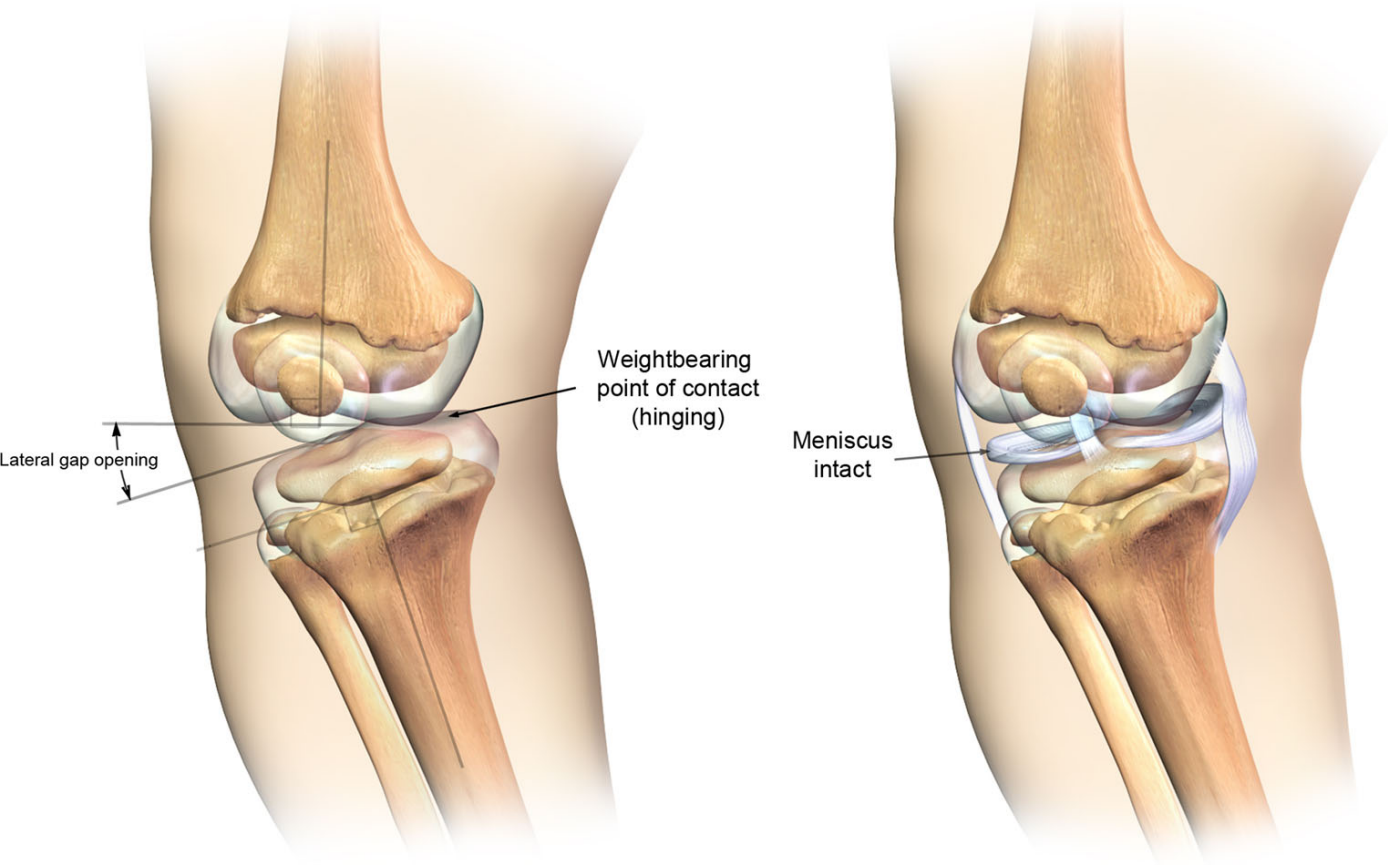
Illustration of osseous and cartilaginous abnormalities in frontal view

The metaphyseal alterations in the magnified illustration (Fig. 5) show a convoluted pattern of the bony metaphysis and junctural physis. Fibular overgrowth and joint laxity and genu varum coexisted but causation could not be established in this review. Computer reconstructions of the cartilaginous distal femoral condyles demonstrate a weight-bearing point of contact on the medial tibial plateau while the lateral portion of the knee shows an opening gap. Online Resource 1 depicts a rotating 3D computer model of Fig. 5.

**Fig. 6:**
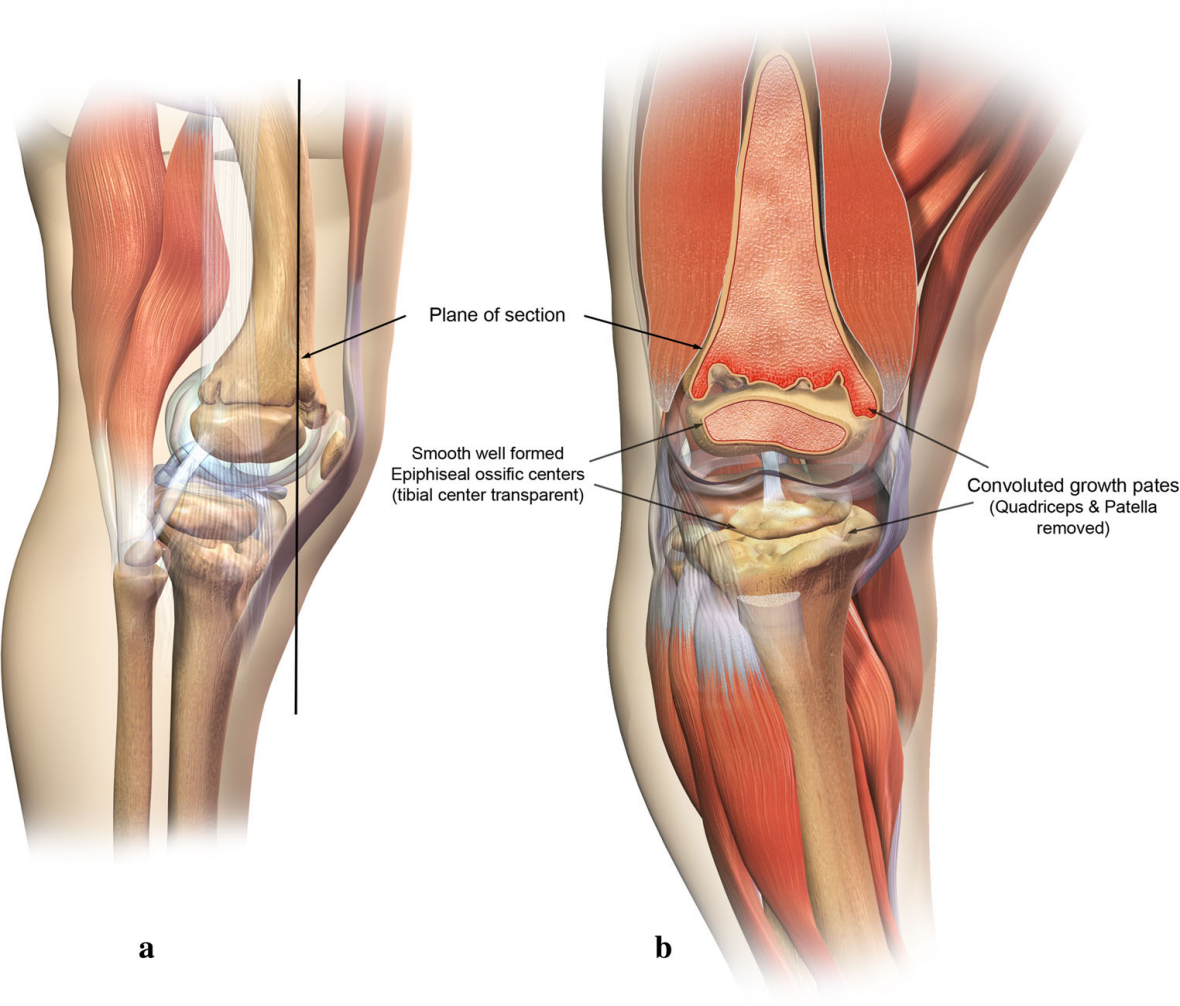
**a** Lateral view illustration of typical knee anatomy in cartilage hair hypoplasia. **b** Anterior illustration of typical knee anatomy in cartilage hair hypoplasia (patella removed)

The convoluted pattern of the bony metaphysis likely reflects irregular cartilaginous invaginations into the ossification zone of the metaphysis from a disordered maturation process within the physis (Fig. 6a, b). Online Resource 2 depicts a rotating 3D computer model of Fig. 6.

Likewise, even in the face of excessive laxity of the joints of the hand and concomitant shortness, we did not encounter any clinically significant complaints. Inasmuch as a large number of patients were Old Order Amish (65 patients), the usual types of employment probably correlate with the lack of complaints (blacksmith, farmer, lumber work, rough and finish carpenter).

## Conclusion

The spectrum of orthopaedic manifestations typically and frequently seen in CHH has been elucidated in this paper based on this study of the largest group of patients so far reported in the North American literature. Thirty-four percent of 95 patients with the condition had undergone surgical realignment osteotomies of the long bones of the lower extremities.

## Electronic supplementary material

Below is the link to the electronic supplementary material.

Supplementary material 1 (MPG 7216 kb)

Supplementary material 2 (MPG 6248 kb)
